# The natural history of incidental retroperitoneal schwannomas

**DOI:** 10.1371/journal.pone.0215336

**Published:** 2019-04-15

**Authors:** Akira Ogose, Hiroyuki Kawashima, Hiroshi Hatano, Takashi Ariizumi, Taro Sasaki, Tetsuro Yamagishi, Naoki Oike, Syoichi Inagawa, Naoto Endo

**Affiliations:** 1 Department of Orthopedic Surgery, Uonuma Institute of Community Medicine, Niigata University Medical and Dental Hospital, Minami-Uonuma, Niigata, Japan; 2 Division of Orthopedic Surgery, Niigata University Graduate School of Medical and Dental Sciences, Chuo-ku, Niigata City, Niigata, Japan; 3 Department of Orthopedic Surgery, Niigata Cancer Center Hospital, Kawagishicho, Chuo-ku, Niigata City, Niigata, Japan; 4 Division of Radiology, Niigata University Graduate School of Medical and Dental Sciences, Chuo-ku, Niigata, Japan; BIDMC, UNITED STATES

## Abstract

The natural history of asymptomatic retroperitoneal schwannomas is poorly understood. This study aimed at investigating the natural history of incidental retroperitoneal schwannomas. The medical charts and imaging studies of 22 asymptomatic patients under observation for at least 12 months for retroperitoneal schwannomas were reviewed. The duration of follow-up ranged between 13 and 176 months (mean 48 months). In the 22 patients managed by the “wait and see” approach, the average tumor size at initial presentation was 51 mm, which increased to 57 mm at final follow-up. During the final follow-up, 2 patients required surgical treatment for tumor enlargement, while the remaining patients remained asymptomatic without surgery. The average growth rate of the tumors was 1.9 mm/year (range: -1.9 to 8.7 mm/year). The majority of asymptomatic retroperitoneal schwannomas demonstrate minimal growth and may be suitable for management with the “wait and see” approach.

## Introduction

Retroperitoneal schwannomas are rare, comprising between 1 and 3% of all schwannomas [1]. Surgical removal is the mainstay of treatment, offering good prognosis in the event of complete removal [1–6]. However, surgery is an invasive procedure; extensive resections are associated with a higher risk of complications, which may necessitate prolonged hospitalization [1–6]. Retroperitoneal schwannomas are rarely symptomatic [6] and there are several short-term reports of case series that were managed without surgery [7–10]. Although rare, malignant peripheral nerve sheath tumors also develop in the retroperitoneum. Careful imaging and core needle biopsy are important for differential diagnosis between benign and malignant tumors in the retroperitoneum [11]. The long-term natural history of retroperitoneal benign schwannomas is largely unknown. The present study retrospectively investigated the management of retroperitoneal schwannomas with the “wait and see” policy, and studied the suitability of this approach for asymptomatic cases.

## Patients and methods

A total of 38 patients were diagnosed with retroperitoneal schwannomas at our institute between 2000 and 2018. Among them, 16 underwent surgical treatment immediately after referral owing to prolonged sciatic pain or numbness in the lower extremity, or because they were suitable candidates for surgery at initial presentation. There were 7 males, and 9 females. The mean patient age was 56 years (range: 37 to 70). All tumors were removed by enucleation via anterior retroperitoneal approach without any complications (S1 Table).

Follow-up with observation (wait and see approach) was selected for 22 patients with schwannomas that were diagnosed either by biopsy or by the typical diagnostic findings on imaging including the target sign or central enhancement [11]. The diagnosis was confirmed by computed tomography (CT) guided needle biopsy in 15 cases, while the remaining 7 were diagnosed by the typical findings on imaging. All biopsied specimens were diagnosed by the senior pathologists with immunohistochemical confirmation of strong and diffuse staining for S-100 protein. The clinical data of these 22 patients comprising 13 males, and 9 females, were retrospectively analyzed. The mean patient age was 57 years (range: 33 to 75). The average observation period was 48 months (range: 13 to 176 months) (Table 1). The patients were evaluated by CT or magnetic resonance imaging (MRI) between every 6 months to every other year, and surgical treatment was considered when symptoms appeared. The maximum diameter of the tumor in the axial, coronal, or sagittal section was recorded as the diameter of the tumor. In this series, there was no patients with neurofibromatosis (NF) 1, NF2, or schwannomatosis.

**Table 1:** Patient and tumor characteristics.

No	age	sex	Cause of initial imaging study	Biopsy	Location	Follow-up period (months)	Baseline diameter (mm)	Final diameter (mm)	Growth rate (mm/yr.)
1	60’s	M	Follow-up after surgery of colon cancer	Yes	Lumbo-sacral plexus	176	60	70	0.7
2	60’s	M	Hematuria	Yes	L3 root	112	36	45	1
3	30’s	F	Transient low back pain	No	L5 root	92	19	45	3.4
4	50’s	F	Genital bleeding	Yes	Lumbo-sacral plexus	90	58	54	-0.5
5	70’s	F	Hematuria	No	Lumbo-sacral plexus	76	46	58	1.9
6	40’s	M	Follow-up after surgery of gastric cancer	Yes	Lumbo-sacral plexus	60	38	35	-0.6
7	70’s	F	Follow-up after surgery of breast cancer	Yes	L4 root	51	57	83	6.2
8	60’s	M	Ultrasonography for general checkup	Yes	S1 root	50	94	102	2.2
9	70’s	F	Transient low back pain	Yes	S1 root	38	73	78	1.6
10	50’s	M	Transient buttock pain	Yes	L5 root	34	15	15	0
11	50’s	M	Ultrasonography for general checkup	No	L2 root	33	32	42	3.7
12	50’s	F	Ultrasonography for general checkup	No	Lumbo-sacral plexus	33	25	24	-0.2
13	50’s	M	Ultrasonography for general checkup	Yes	Lumbo-sacral plexus	29	64	71	2.9
14	40’s	F	Transient low back pain	Yes	Lumbo-sacral plexus	26	50	46	-1.9
15	50’s	M	Ultrasonography for general checkup	No	L2 root	26	65	71	2.8
16	60’s	M	Transient low back pain	Yes	S1 root	25	58	64	2.9
17	60’s	M	Transient low back pain	Yes	L3 root	23	60	60	0
18	30’s	F	Ultrasonography for general checkup	Yes	L3 root	20	45	64	8.7
19	40’s	M	Ultrasonography for general checkup	No	L2 root	18	69	84	3.5
10	50’s	M	Ultrasonography for general checkup	Yes	Lumbo-sacral plexus	14	57	58	1.4
21	70’s	F	Ultrasonography for general checkup	Yes	L4 root	14	55	57	2.9
22	40’s	F	Ultrasonography for general checkup	No	L2 root	13	36	36	0

This study was approved by the Niigata University Hospital Institutional Review Board (IRB).

## Results

Table 1 shows the characteristics of the follow-up cases. No patient complained of sustained lower extremity pain, or sensory or motor dysfunction at the first visit. The tumor was detected by abdominal ultrasonography examination during routine general investigations in 10 patients, and by MRI in 6 individuals while investigating for temporary low back pain. The remainder were diagnosed by CT, during follow-up imaging for different cancers, investigation for hematuria, and investigation of genital bleeding in 3, 2, and 1 cases, respectively.

Eight tumors arose from the lumbo-sacral plexus, 4 were from the L2 root, 3 each from the L3, and S1 roots, and 2 each from the L5 and L4 roots. In 22 patients who were under follow-up from the first visit, an increase in the size of tumors were noted in 16 cases, while a minor reduction was observed in 3 cases. No change in tumor size was noted in the remaining 3 cases.

**Fig 1:**
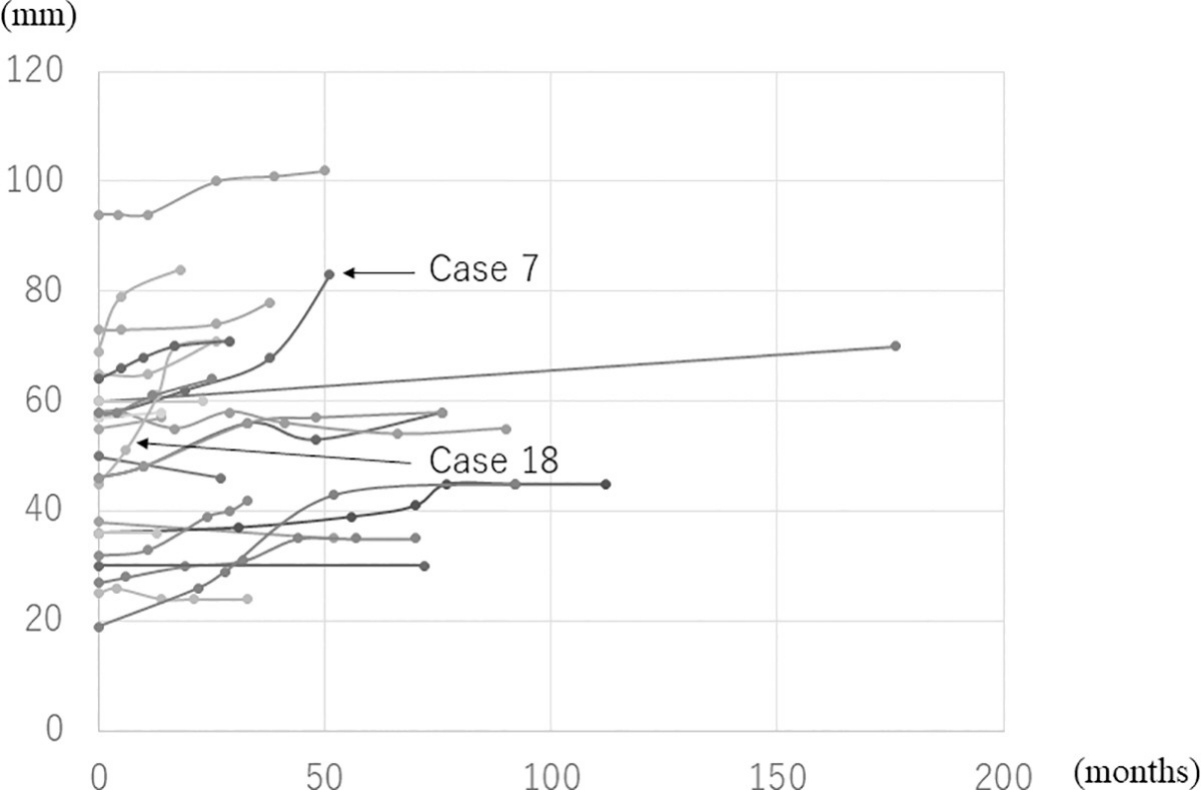
Change in diameter of the schwannomas in 22 patients during the follow-up period.

**Fig 2:**
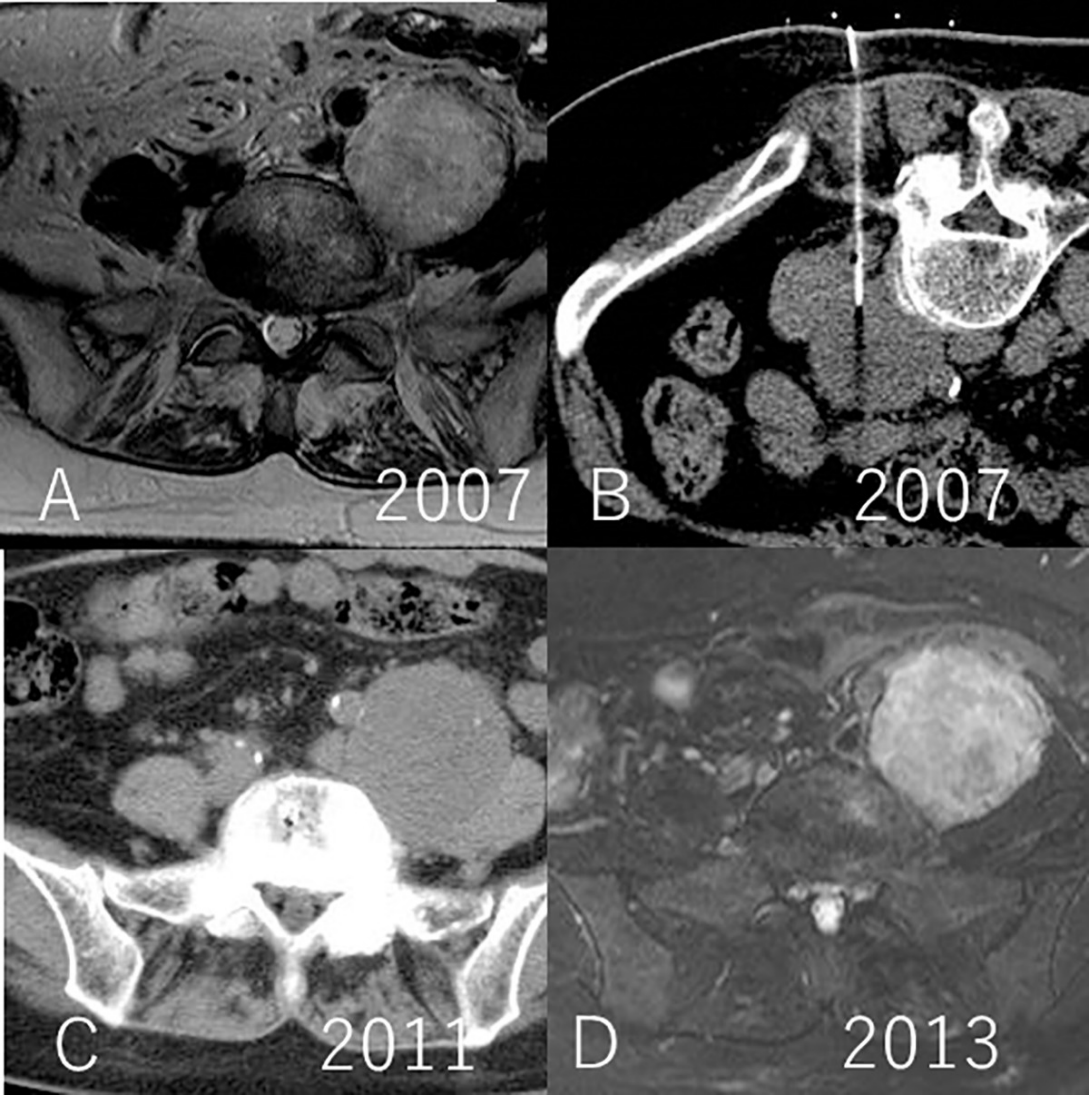
CT and MRI. A. Initial T2 weighted MR image in 2007; B. CT guided needle biopsy in 2007; C. CT scan in 2011; D. T2 weighted MR image with fat suppression in 2013 (case no. 5). Base line diameter of the tumor was 46 mm, and final diameter of the tumor was 58 mm.

**Fig 3:**
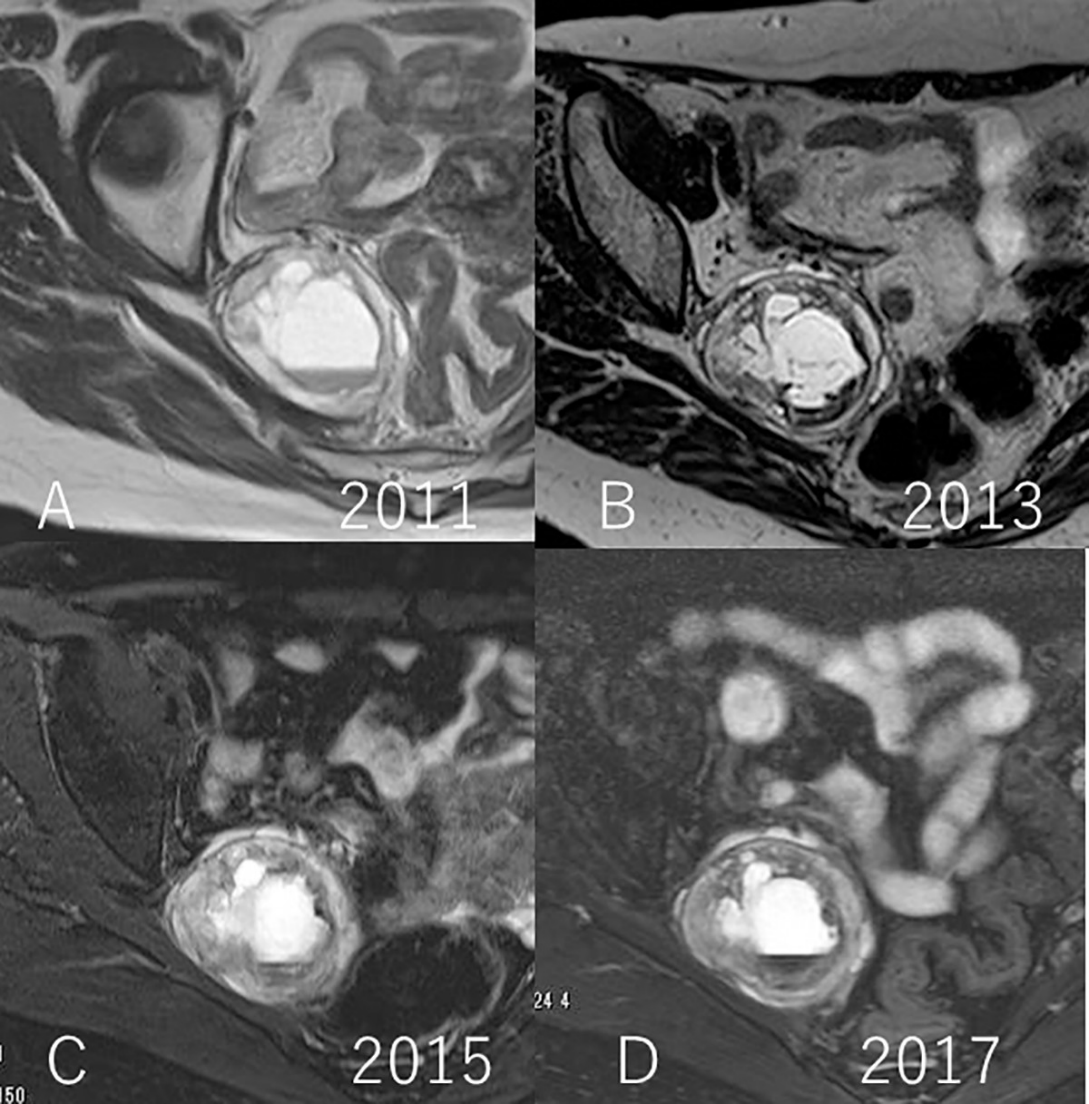
A. Initial T2 weighted MR image in 2011; B. T2 weighted MR image in 2013; C. T2 weighted MR image in 2015; D. T2 weighted MR image in 2017 (Case no. 4). These images show cystic change. Base line diameter of the tumor was 58 mm, and final diameter of the tumor was 54 mm.

**Fig 4:**
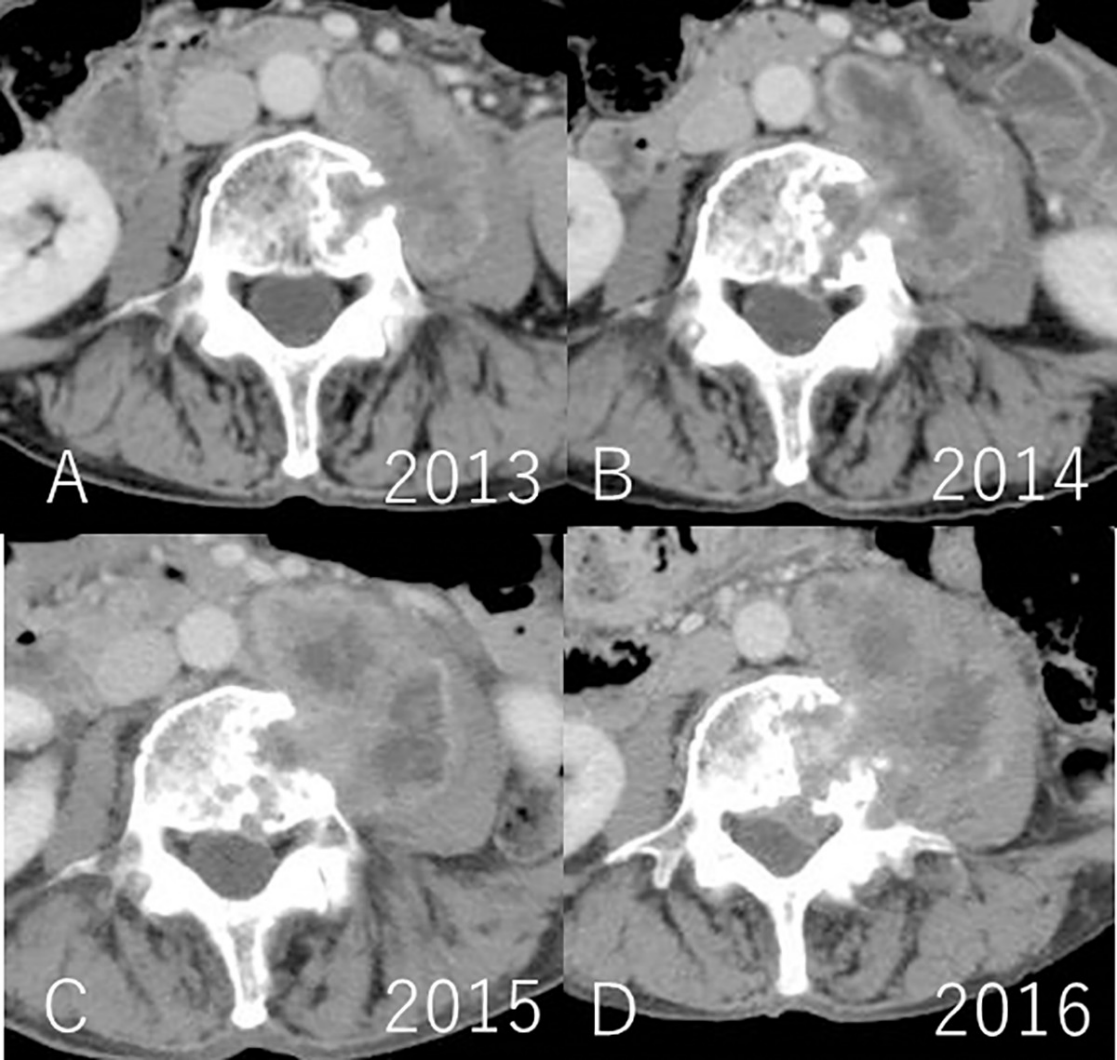
CT scan following contrast injection in 2013; B. CT scan following contrast injection e in 2014; C. CT scan following contrast injection in 2015; D. CT scan following contrast injection in 2016 (Case no.7). Base line diameter of the tumor was 57 mm, and final diameter of the tumor was 83 mm. Finally the tumor was surgically removed with bone graft and spinal instrumentation.

The average tumor diameter at the beginning of follow-up was 51 mm (range: 15 to 94 mm), which increased to 57 mm (range: 15 to 102 mm) at completion. Two cases, namely numbers 7, and 18, underwent surgery owing to an increase in the size of the tumors at 57 months and 18 months after initial consultation, respectively. The tumor growth rate in the individual cases was 6.2 and 8.7 mm/year. Surgical treatment was not required in 20 patients in this cohort, as they remained asymptomatic throughout the follow-up period. The average cumulative tumor growth rate in this cohort was 1.9 mm/year (range: -1.9 to 8.7 mm/year) (Figs 1, 2, 3 and 4) (S1 File)

## Discussion

Retroperitoneal schwannomas cause abdominal distension, low back pain, lower limb pain and sensory and motor disturbances in the lower extremities [1–6, 11,12]. However, it is not uncommon for asymptomatic tumors to be incidentally discovered, a trend that has become increasingly common for these tumors with advances in imaging. Strauss et al. reported that 13 of the 28 retroperitoneal schwannomas in their cohort were asymptomatic [6], while Li et al. reported that in 12 out of 82 cases in their series, the tumors were incidentally detected [5]. Among the 38 patients who visited our facility, 16 patients had symptoms that were apparently related to the tumor, necessitating immediate surgery, while 22 were asymptomatic. The high ratio of asymptomatic patients in this cohort may represent the trends of this disease in Japan. Ultrasonography examinations of the abdomen and pelvic cavity are often performed during general investigations in Japan. In this series, the tumor was detected during ultrasonography in 10 of 22 cases. There is no established guideline on the optimal treatment of asymptomatic retroperitoneal schwannomas.

Various reports on the conservative management of vestibular schwannomas are available in literature. Kichman et al. reported that in their cohort, 37% of vestibular schwannomas exhibited tumor growth and 15% required invasive treatment during the 10-year follow-up period [13]. Zou et al. noted that conservative management using the “wait and scan” method was suitable for 87% of vestibular schwannomas [14]. Surgery is therefore not currently recommended immediately after tumor diagnosis in asymptomatic vestibular schwannomas [13,14].

Conservative management has also been described for spinal schwannomas. Ozawa et al. reported that only 1 of 6 cases in their series of spinal cord schwannomas required surgery during 3 to 10 years of follow-up [15]. The average tumor growth rate in their series was 1.8 mm/year (range: 0 to 7 mm/year). This growth rate was similar to that of the present study cohort. Lee et al. reported that 6 of 56 spinal intradural schwannomas in their series needed surgical treatment during a mean follow-up of 43.6 months [16].

Published literature on the natural history of retroperitoneal schwannomas is scarce. One of the probable reasons is that retroperitoneal nerve sheath tumors tend to be large, and in certain instances, degeneration including intralesional cystic changes occurs, making distinction from sarcoma difficult [10–12]. Although essentially benign, some cases do may have clinical consequences. Symptoms can include neuropathic pain, voiding symptoms, hydronephrosis due to compression by the tumor etc. [10–12], and follow-up is therefore necessary. In our practice, periodic imaging is performed to monitor changes. In recent years, endoscopic or robotic minimally invasive surgery is also being employed for the management of retroperitoneal schwannomas, with an acceptable rate of surgical complications [17–19]. Ji et al. reported complication rates of 12.5% with laparoscopic resection of retroperitoneal schwannomas [18], while Liu et al. reported complication rates of 12% with robotic resections of benign retroperitoneal tumors, including schwannomas [19].

In the present study, only 2 of 22 patients needed surgery during follow–up, and the remaining 20 patients experienced no worsening of symptoms during the 13 to 164 months of follow-up.

Since the number of cases in this study is small, it is unclear which cases grow rapidly and produce symptoms. It will be a further problem whether there is a difference in the tumor growth speed between the cases with symptoms and the cases without symptoms

The majority of asymptomatic retroperitoneal schwannomas showed very slow growth rates. Three tumors showed minor reductions in size during follow-up. The growth rates of asymptomatic schwannomas cannot be compared directly with those of their symptomatic counterparts. The “wait and see” policy is suitable for asymptomatic retroperitoneal schwannomas. This approach is often adopted for many benign tumors, including incidental meningiomas, and desmoid tumors [20,21].

The possibility of malignancies including malignant peripheral nerve sheath tumors, leiomyosarcomas, and dedifferentiated liposarcomas should be considered in the event of rapid tumor growth. Central enhancement pattern, target sign in imaging studies strongly suggest benign schwannoma. In contrast severe motor weakness, progressive pain, and rapidly enlarging tumoral mass suggest malignant lesions [11].

In conclusion, the average growth rate of asymptomatic retroperitoneal schwannomas in the present cohort was 1.9 mm/year (range: -1.9 to 8.7 mm/year), with few patients needing surgical treatment during the period of follow-up. The “wait and see” approach is suitable for management of this tumor, and maybe considered in cases that are asymptomatic at presentation.

## Supporting information

S1 Table Table of surgically treated cases. (DOCX)

S1 File Change of tumor size. (XLSX)
