# Supplementary material for: The natural history of incidental retroperitoneal schwannomas
Source: PLoS One. 2019 Apr 15;14(4):e0215336. doi: 10.1371/journal.pone.0215336 (PMC6464223; doi:10.1371/journal.pone.0215336)
Supplement: S1 Table — (DOCX) [file pone.0215336.s001.docx]

| Table . Patient and tumor characteristics of surgically treated cases at presentation | | | | | |  |  |  |  |  |  |
| --- | --- | --- | --- | --- | --- | --- | --- | --- | --- | --- | --- |
| No | age | sex | Cause of initial imaging study | Location | Baseline diameter (mm) | preoperative paralysis | postoperative paralysis |  |  |  |  |
| 1 | 50's | M | Thigh pain | L4 root | 90 | none | none |  |  |  |  |
| 2 | 60'S | F | Thigh pain | L4 root | 80 | none | none |  |  |  |  |
| 3 | 30'S | M | Low back pain | Lumbo-sacral plexus | 140 | none | none |  |  |  |  |
| 4 | 50'S | F | Low back pain | Lumbo-sacral plexus | 100 | none | none |  |  |  |  |
| 5 | 60's | F | Low back pain | Lumbo-sacral plexus | 80 | none | none |  |  |  |  |
| 6 | 50's | M | Low back pain | Lumbo-sacral plexus | 90 | none | none |  |  |  |  |
| 7 | 50's | M | perianal pain | Lumbo-sacral plexus | 160 | MMT4 | MMT4 |  |  |  |  |
| 8 | 60's | F | buttock pain | Lumbo-sacral plexus | 170 | MMT4 | MMT4 |  |  |  |  |
| 9 | 70's | F | buttock pain | Lumbo-sacral plexus | 120 | MMT4 | MMT4 |  |  |  |  |
| 10 | 40's | F | buttock pain | Lumbo-sacral plexus | 60 | MMT4 | MMT4 |  |  |  |  |
| 11 | 60's | F | buttock pain | Lumbo-sacral plexus | 55 | none | none |  |  |  |  |
| 12 | 40's | M | Ultrasongraphy for general checkup | L4 root | 69 | none | none |  |  |  |  |
| 13 | 40's | M | Thigh pain | L4 root | 41 | none | none |  |  |  |  |
| 14 | 60's | F | buttock pain | Lumbo-sacral plexus | 26 | none | none |  |  |  |  |
| 15 | 60's | F | back pain | L3 root | 160 | none | none |  |  |  |  |
| 16 | 40's | M | Low back pain | Lumbo-sacral plexus | 30 | none |  |  |  |  |  |
|  |  |  |  |  |  |  |  |  |  |  |  |
